# Abandonment or overreach: the ethical failures of health and social systems and the need for contributory reciprocity

**DOI:** 10.3389/fpubh.2026.1797875

**Published:** 2026-06-25

**Authors:** J. R. Baker

**Affiliations:** 1 Faculty of Health, Southern Cross University, Lismore, NSW, Australia; 2 Primary and Community Care Services, Thornleigh, NSW, Australia; 3 Australian Social Prescribing Institute of Research and Education, Surry Hills, NSW, Australia

**Keywords:** agency, contributory reciprocity, ethical abandonment, ethical overreach, quality of life, shared life, social health, social prescribing

## Abstract

Despite rising investment in health and social services, loneliness, mental distress, and social disconnection continue to worsen across high-income countries. This pattern suggests the challenge may not lie solely in insufficient provision, but in how systems conceptualize people and value. Within increasingly individualized and consumption-oriented contexts, people are often positioned as recipients of services rather than contributors to social and civic life, with value reduced to economic or service-based metrics rather than relational and civic contributions.

These assumptions shape system design, giving rise to two recurring patterns of failure. In ethical abandonment, systems retreat into markets and transactions, positioning people as consumers without preserving conditions for meaningful agency or contribution. In ethical overreach, systems use prescriptive, behavioral, or medicalised approaches that substitute institutional control for personal authorship, positioning people as objects to be optimized. Although these approaches appear opposed, both deny that people can matter to others beyond their consumption of support, undermining reciprocity, eroding shared life, and driving system expansion.

*Contributory reciprocity* is proposed as an alternative ethical orientation, recognizing people as potential contributors to shared life, not merely recipients of support. It partitions responsibility: systems secure real opportunity and preserve civic space; individuals retain authorship over engagement. The framework maintains expectation, rather than obligation, that people can create value for others, rejecting the drift toward lives organized around consumption alone.

Social prescribing is examined as a practice through which contributory reciprocity can be enacted. When practiced as invitation rather than prescription, it renders opportunities visible, lowers barriers to entry, and returns authorship to individuals. When reduced to behavioral direction or commodified experience, it risks reproducing the consumption-oriented logic it seeks to address. Actionable recommendations are provided across system design, commissioning, evaluation, and policy development, emphasizing opportunity over activity delivery, relational work, and distinguishing invitation from uptake.

## 1 Introduction

Across high-income welfare states, health and social systems are under sustained pressure to respond to rising loneliness, mental distress, disability, and social disconnection. These challenges are increasingly recognized as structural threats to population health, social cohesion, and system sustainability (1, 2). In response, governments and health systems have expanded services, diversified interventions, and turned toward social and community-based approaches. Yet despite this activity, a persistent tension has been widely observed. Support is delivered, resources are consumed, and outcomes are measured, while indicators of loneliness, social disconnection, and mental distress remain stubbornly elevated across high-income welfare states (1–3).

This tension is now widely acknowledged at the global policy level. Recent World Health Organization work has framed loneliness and social isolation as major public health challenges with consequences comparable to established biomedical risk factors (1, 4). At the same time, decades of work on the social determinants of health have shown that expanding services alone does not reliably translate into improved lived well-being, particularly where social connection, meaning, and participation are weak (3, 5). Health systems appear increasingly active, yet increasingly strained.

Two terms used throughout this paper require brief characterization. *Shared life* is used in the Aristotelian sense of *suzēn:* the conditions under which people live together in ways that are mutually perceiving, reciprocally meaningful, and oriented toward purposes that extend beyond individual consumption (6, 7). *Contribution* is used throughout this paper to denote any voluntary act through which a person's presence, effort, care, or attention creates value for others — whether or not that act is economic, formally recognized, or measurable. This distinguishes it from narrower civic participation definitions that emphasize activity attendance or labor market involvement (8).

Within this frame, the situation can be understood more precisely. Systems have expanded provision and access, yet indicators of loneliness, social disconnection, and mental distress remain elevated. This suggests a misalignment between what systems deliver and the social conditions they are intended to sustain. While services can alleviate need, they do not necessarily create or preserve the conditions under which shared life and contribution can emerge.

One consequence is that people are not consistently positioned as needed by others or able to contribute value beyond their own care or consumption. Where this occurs, opportunities for reciprocity and mutual recognition are limited, and shared life is weakened. As a result, systems often become highly competent at delivering services while remaining unclear about the forms of value and social life they are intended to sustain.

This paper argues that the problem is not primarily one of provision, evidence, or intent, but of ethical framing. Contemporary well-being systems struggle to articulate what they are ultimately trying to sustain. People are alternately treated as subjects of intervention or as consumers of support, while the moral language required to describe shared life, mutual responsibility, and human worth remains underdeveloped. As a result, systems often become highly competent at delivering services without necessarily strengthening the social conditions that sustain meaning, connection, and shared purpose.

This ethical thinning is reinforced by how value is recognized and operationalised. Well-being is frequently assessed through economic, behavioral, or administrative proxies such as service utilization, labor participation, independence, risk reduction, or compliance with recommended behaviors (5, 9). These measures are not without importance. Economic security matters and markets play a vital role in organizing resources, and behavioral change can improve health outcomes. However, when such proxies come to dominate how success is defined, they narrow the moral field within which lives are understood. Human worth and the quality of a life are not reducible to economic productivity, behavioral optimisation, or service consumption.

The limits of this framing are visible even in well-intentioned policy architectures. Major disability and well-being systems frequently define their aims in terms of participation, including social, economic, or community participation (10, 11). While such language signals inclusion, it also reveals a modest ethical ambition. Participation implies presence without necessity, attendance without consequence. It allows people to take part but stops short of recognizing them as contributors whose actions, relationships, and efforts might matter to others.

The distinction is not semantic. A system oriented toward participation asks whether individuals can access activities. A system oriented toward contribution asks a different question: how people might shape the world around them in ways that are meaningful to them and valuable to others. When systems fail to ask this second question, they risk supporting lives organized around consumption rather than shared purpose, particularly for groups already positioned as dependent, such as people living with disability, mental illness, older adults, and unpaid caregivers.

In response to this ethical gap, this paper develops a responsibility-partitioned account of well-being grounded in contributory reciprocity. It argues that systems have a duty to preserve the conditions under which contribution is possible, through access, capability, and the protection of shared civic space, while individuals retain responsibility for how and whether they engage. Social prescribing is examined as an illustrative mechanism that reveals both the promise and the limits of invitational approaches to shared life. While this paper draws on capability approaches, relational ethics, social capital theory, and asset-based community development, contributory reciprocity is distinct from each. It differs from capability approaches in its explicit emphasis on contribution to others rather than individual functioning (12, 13) from co-production frameworks in refusing to instrumentalise contribution as a means of improving service efficiency (14) and from asset-based community development in situating contribution within a responsibility-partitioned ethical framework that also addresses what systems owe individuals (15). These distinctions are developed fully in Section 4.

The aim is not to prescribe a single vision of the good life, nor to moralize individual behavior. Rather, it is to recover an ethical orientation in which people are recognized not merely as recipients of support, but as persons who matter, and whose presence, effort, and contribution can matter to others. This article is a Policy and Practice Review that integrates policy analysis, conceptual ethics, and selected empirical literature to examine how contemporary health and social systems conceptualize participation and contribution. Sources were selected purposively to represent breadth across three domains rather than exhaustiveness within any single one: influential policy frameworks (e.g., WHO, OECD, national disability systems), key theoretical traditions (e.g., capability approach, relational ethics), and illustrative practice examples. The goal is to provide more than a systematic review on empirical evaluation, developing a normative and conceptual framework that can inform policy, design, commissioning, and evaluation. Accordingly, claims about system failures and social erosion are advanced as normative and conceptual propositions informed by convergent evidence, rather than as empirically demonstrated causal findings. Contributory reciprocity is proposed as a corrective analytical lens, not a definitive empirical solution.

## 2 The ethical boundary of responsibility: partitioning system and individual accountability

Any ethical account of well–being must distinguish clearly between what social systems are responsible for providing and what must remain the responsibility of individuals themselves. Without this boundary, systems risk either overreach, attempting to shape lives directly, or abdication, retreating into nominal choice while leaving people unsupported. Both positions undermine dignity and agency in different ways.

This paper adopts a responsibility-partitioned ethical orientation. On this account, systems are responsible for securing the conditions under which people can realistically pursue lives of quality, while individuals retain responsibility for whether and how they act within those conditions. Recognizing individuals as responsible agents is not merely a practical distinction but an ethical one, as it affirms their moral standing as persons whose choices and actions can matter to others (12, 16).

### 2.1 What systems owe individuals

The primary obligation of social systems is not to shape lives directly, but to secure the conditions under which people can realistically pursue lives of quality. This obligation is best understood in terms of preserved opportunity: the sustained existence of accessible pathways into shared civic, cultural, and social life that remain open for people to enter, explore, and use without excessive friction, cost, or gatekeeping.

Opportunity in this sense is not merely formal or programmatic. It is ethically inadequate if it exists only on paper, or behind bureaucratic complexity, cultural barriers, or market thresholds that render it unusable in practice. What counts as accessible, however, is not uniform. Barriers to entry are experienced differently across individuals and contexts, meaning that opportunity must be responsive to variation in capability, circumstance, and need rather than defined by a single threshold. Systems therefore have a responsibility to provide the material, social, and informational conditions that make engagement realistically possible. This includes basic material security such as access to food, housing, healthcare, and safety, but also extends to education, information, social connection, navigational support, and the preservation of shared civic and community spaces (3, 12, 13).

Finally, systems bear responsibility for removing unjust barriers. Structural disadvantage, discrimination, and institutional fragmentation can render nominal opportunity meaningless. Where such barriers are systemic in origin, responsibility for addressing them lies with the system rather than the individual (13). These obligations are substantial and demanding. They require investment, coordination, and ethical intent, but they do not extend indefinitely. Once opportunity is real, accessible, and usable, responsibility for whether and how to engage must remain with individuals themselves.

**Figure 1:**
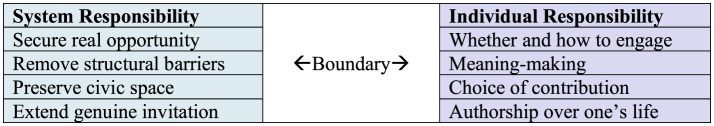
Responsibility partitioning in contributory reciprocity.

In practice, this distinction is visible in social prescribing: a system fulfills its obligation by ensuring a link worker can connect someone to local community activities, removing informational and navigational barriers to entry. Where opportunity is genuinely accessible, responsibility for whether and how to engage rests with the individual. This boundary is summarized in Figure 1.

### 2.2 Where system responsibility ends

The ethical significance of this boundary lies in what falls beyond it. Responsibility for action, engagement, and meaning-making cannot ethically be transferred to institutions. Motivation cannot be supplied by systems without coercion or manipulation, because to do so would treat individuals as means to system goals rather than as authors of their own lives (17). Meaning cannot be imposed without eroding autonomy. Volition, the act of choosing whether and how to engage, remains irreducibly personal.

This boundary is frequently blurred in health and social systems, particularly when outcomes are prioritized without sufficient attention to agency. When individuals do not engage with available opportunities, systems may be tempted to interpret non-uptake as failure, resistance, or pathology. Such interpretations risk collapsing responsibility entirely onto the system, thereby justifying increasingly intrusive forms of intervention. This paper rejects that move. Outcome differences that arise after fair opportunity has been provided are not, in themselves, moral failures of the system. Respect for agency requires accepting that individuals may decline, disengage, or choose differently, even where systems believe engagement would be beneficial.

### 2.3 Human worth and the moral preconditions of responsibility

Establishing a boundary between system and individual responsibility is necessary but insufficient. Assigning responsibility back to individuals is itself an ethical act, and it rests on assumptions about what people are capable of doing with that responsibility. That boundary is only ethically meaningful if systems are built on the belief that people matter and are capable of exercising agency and contributing value to others. Where systems assign responsibility while presuming incapacity in practice, they act in bad faith: they invoke the language of agency while designing for dependence. The ethical question is therefore not only where responsibility ends, but whether systems genuinely believe in the agency they claim to respect.

The most fundamental responsibility of social systems is to operate on the premise that all people matter. Not conditionally, not instrumentally, and not only when they contribute in ways systems can measure, but inherently and unconditionally, by virtue of being human. Belief here is not satisfied by statements of intent, values, or rights. It is tested by whether systems are designed as though people are genuinely capable of acting, choosing, learning, and contributing in some form. Where responsibility is formally allocated but practically neutralized through risk aversion, over-specification, substitution of judgement, or permanent mediation, claims about dignity and agency become performative rather than real.

Systems do not exist independently of the people who enact them. Policies and procedures are applied through professional judgement, organizational culture, and everyday decision-making. Where it is safer, easier, or more defensible to treat people as incapable than as capable, that assumption shapes how systems actually function. In such cases, disbelief in people's capacity is not incidental; it is doing the work of the system. Recognizing capability does not require assuming full cognitive capacity, independence, or productivity. Capacity is not all-or-nothing, and contribution is not synonymous with productivity or economic output. Even where agency is constrained by age, illness, changes in cognition, or disability, people may still contribute through presence, familiarity, care, emotional resonance, or relational continuity. Ethical failure lies not in acknowledging limits, but in treating limitation as the absence of value altogether.

If systems do not believe that all people have worth, capacity, and potential, or that they can learn, grow, contribute, and matter to others, then no amount of service provision, evidence, or investment will sustain shared life. Evidence from high-income welfare contexts suggests that, without this orientation, systems can drift toward managing people as problems or maintaining them as dependents, consistent with broader accounts of declining social capital and civic life (18, 19).

#### 2.3.1 Trusting people with complexity

Belief in people means, first, believing they are capable of reasoning, learning, and making sense of their lives. This is not the same as assuming people will always choose optimally, that information alone solves problems, or that everyone has equal access to knowledge. It means refusing the presumption of incompetence. It means trusting people with real information, including complexity, uncertainty, and risk, rather than managing them toward compliance (20). It means designing systems that treat individuals as capable of understanding their circumstances, weighing options, and making choices that reflect their values, even when those choices diverge from what systems recommend. Where stakes are high or uncertainty is difficult to tolerate, systems can drift from trust toward control, narrowing the space for plural reasoning and substituting adherence for partnership.

Trusting people with complexity does not mean abandoning collective responsibility. It means maintaining transparency, epistemic humility, and respect for people's capacity to reason under uncertainty, not only in their own interests, but as members of a shared community whose choices and contributions matter to others (21). When systems lose this belief, the risk is not simply poor communication, but a deeper ethical collapse: citizens and professionals alike are treated less as reasoning agents and more as objects of behavioral management.

#### 2.3.2 Designing for growth, not stasis

Second, believing that all people matter means believing people can grow, change, and become more than they currently are. This belief refuses to reduce individuals to their present circumstances, diagnoses, or limitations. It resists the temptation to define people by what they cannot do, or by the risks they present, or by the services they consume. It holds open the possibility that people can learn new things, recover capabilities thought lost, surprise themselves and others, and exceed the expectations placed on them (13, 22).

This is not naive optimism. People face constraints, like illness, disability, trauma, disadvantage and/or age. Growth is not linear, universal, or guaranteed. But systems that do not believe growth is *possible* organize themselves around maintenance rather than invitation. They design for stasis rather than emergence (23). They ask, “what do you need?” but never “what might you become?” or “how might you matter to others?” In doing so, they quietly signal that nothing more is expected, and that the person they see now is all they will ever be.

#### 2.3.3 Recognizing capacity to contribute

Third, it means believing people can contribute to shared life in ways that matter. This is the belief most absent from contemporary health and social systems. When people are positioned primarily as recipients, consumers, patients, or service users, systems implicitly answer the question, “what are you here for?” with, “to be supported, to consume resources, or to comply with interventions.” Contribution, if acknowledged at all, is treated as bonus, optional, or reserved for those deemed “recovered” or “capable.”

Rather than generosity, this is abandonment dressed as care. To never ask how someone might contribute is to deny them recognition as a person whose presence, effort, and care could matter to others (18). It is to assume that they have nothing to give, no role to play in shared life beyond passive receipt. It collapses human worth into need and treats capacity as exceptional rather than assumed.

The ethical alternative requires systems to assume, structurally and operationally, that all people can contribute something, even if that contribution bears little resemblance to paid work, formal volunteering, or measurable output (24, 25). Contribution may take many forms: presence, care, encouragement, creativity, reliability, humor, attention, recognition, emotional connection, reassurance, memory, affection, or simply showing up. Economic or labor utility should not be confused with human contribution or the capacity to matter to others. Even where people communicate, participate, or relate to others in highly constrained, unconventional, or non-verbal ways, their presence, recognition, responsiveness, and relational connection may still carry profound meaning to others. The ethical failure lies not in recognizing human vulnerability or dependence, but in presuming that these extinguish the possibility of mattering altogether.

Belief in contribution is therefore not attitudinal but operational. It is visible in whether systems presume capability in their design, or organize around its absence. The ethical failure is not that contribution goes unmeasured, but that it is never expected or invited. When systems fail to extend this possibility, they risk drifting toward permanent management or dependence. What matters is not the form or quantity of contribution, but the recognition of capacity and the extension of invitation. A system that never invites contribution has already assumed incapacity.

#### 2.3.4 Belief as operational premise

Importantly, believing that all people matter does not mean expecting constant evidence of growth or visible proof of contribution. Belief is not conditional on performance. People who are unwell, exhausted, grieving, incapable, or simply choosing not to engage at a given moment do not forfeit their worth. Systems that demand demonstrable output as proof of value have already abandoned belief, or, at a minimum, trust and hope.

Rather, belief functions as an orientation toward possibility (26). It means organizing systems as though people are capable, even when current circumstances suggest otherwise. It means extending invitations without prescribing uptake. It means providing information that assumes people can think, rather than messages designed to manipulate behavior (27). It means protecting civic space where contribution is possible, even when it is not immediately visible.

The opposite of belief is not skepticism or realism; it is commodification (28). When systems relate to people only through what can be measured, monetised, or administered, they signal that unmeasurable forms of worth, such as care, presence, reliability, and creativity, do not count. Human value collapses into economic value. Contribution becomes invisible unless it generates data. Agency disappears unless it aligns with system goals.

Systems cannot create belief, but they can destroy it. When systems treat people as incapable of reasoning, growth, or contribution, those assumptions become structural reality: people internalize the message, communities organize around it, and shared life thins (18, 19).

The ethical task, then, is not to prove that all people are capable, but to refuse the assumption that they are not. This refusal must be embedded in system design, commissioning, evaluation, and everyday practice. It is the precondition for everything else this paper proposes. Without belief in people, in their capacity to reason, grow, and contribute, contributory reciprocity becomes empty rhetoric, and health and social systems become mechanisms of maintenance rather than invitations to shared life.

## 3 Assessment of current policy approaches: two ethical failures in contemporary health and social systems

The argument of this paper rests on a simple moral claim: that health and social systems fail when they organize support entirely around people *taking* services rather than *giving* to others. When systems stop expecting that people can create value beyond their own consumption and when contribution is treated as exceptional rather than assumed, human worth narrows to a single dimension: the efficient receipt of support. It is a profound ethical failure with structural consequences rather than a neutral administrative choice.

The dual failures examined in this section, both ethical overreach and ethical abandonment, are not separate problems. They are convergent expressions of this single foundational error. Both position people as objects rather than subjects. Both deny that individuals can matter to others through their presence, effort, or care. Both substitute system-determined goals (whether behavioral optimisation or market consumption) for person-authored contribution. And both drive system expansion without natural limit, because consumption-based models have no endpoint: there is always more to deliver, more to optimize, more to purchase. Systems do not have unlimited resources with which to secure diminishing or adverse returns.

When the ethical boundary of responsibility is blurred, and when systems lose belief in people's capacity to reason, grow, and contribute, health and social systems tend to fail in one of two predictable ways. They either extend responsibility too far, attempting to shape lives directly, or retreat too far, withdrawing responsibility under the guise of choice and market provision. While these failures appear opposed, both erode agency, dignity, and shared life. These failures arise from an unresolved ethical tension: how to support people without controlling them, and how to respect choice without abandoning responsibility. When this tension is left unexamined, systems default either to prescription or to transaction.

**Figure 2:**
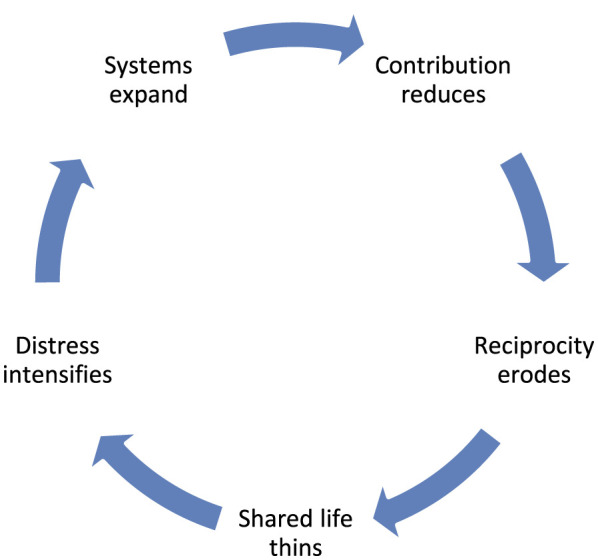
Contribution–consumption cycle.

This paper proposes a conceptual cycle (see Figure 2) as a heuristic rather than an empirically validated causal model: where contribution is absent, reciprocity is likely to erode; where reciprocity erodes, shared life may thin; where shared life thins, isolation and distress tend to intensify; and where distress intensifies, systems expand further, potentially reinforcing patterns of provision and consumption over time.

Convergent evidence on loneliness, social disconnection, and service expansion across high-income contexts is consistent with elements of this pattern (1, 3, 19). However, the cycle is advanced here as a conceptual proposition requiring empirical investigation rather than as a settled causal account. If this dynamic holds, the mechanisms designed to support well-being risk undermining the social conditions on which well-being depends.

This section examines how this failure operates in practice.

### 3.1 Ethical overreach: people as subjects of control

Ethical overreach occurs when systems move beyond enabling opportunity and into prescribing how people ought to live. This represents the first expression of treating people as objects: objects to be optimized, managed, and shaped toward institutional goals rather than recognized as authors of their own lives. When systems assume authority over what constitutes a *good life*, they deny that individuals can create value through their own choices and contributions. Instead, value is defined by compliance with prescribed behaviors, adherence to evidence-based norms, or alignment with population-level outcomes.

This error is now embedded in many contemporary well-being and healthcare approaches. Evidence, risk reduction, and behavioral effectiveness are routinely treated not only as informative but as morally directive. Individuals are steered, nudged, or managed “for their own good,” while meaningful consent is thinned into procedural compliance. Lives are shaped toward normative trajectories deemed optimal for populations, even where those trajectories do not align with what matters to the person themselves. Systems begin to define acceptable forms of behavior, participation, or well-being, and organize support accordingly. Extending system responsibility beyond the provision of real opportunity and into the shaping of how people ought to live is ethically wrong, even if well-intentioned and common. The argument that follows is not against behavioral support, evidence-based practice, or population-level intervention as such; it is against the specific ethical move by which these become morally directive for individual lives regardless of what matters to the person.

The following boxes present illustrative cases. They are offered as heuristic applications of the conceptual framework rather than as systematic policy evaluations. The aim is to show how the failure modes described above are recognizable in practice, not to provide comprehensive assessments of the policies or systems named.

This trajectory reflects a deeper structural shift. When evidence becomes directive rather than informative, both patients and clinicians are repositioned within the system: patients as subjects of optimisation, and clinicians as agents of protocol. The person's needs, values, and authorship recede, while the clinician's capacity to work with pluralism, uncertainty, and divergence is narrowed by expectations of compliance.

At its limit, this represents a collapse of responsibility. When systems assume authority for defining meaning, motivation, or value, individuals are no longer recognized as authors of their own lives. This reflects a broader form of epistemic overreach, in which professional or institutional knowledge claims authority over plural values and lived experience (29, 30). Responsibility cannot be honored if it is absorbed into institutional control, even where that control is well intentioned.

Systems organized primarily around standardized evidence and compliance may also become increasingly amenable to automation, as judgement is reduced to adherence rather than interpretation. The issue is not the use of evidence or technology, but the erosion of the human elements that both require interpretation, negotiation, and responsiveness to what matters to the person. Where professional practice becomes tightly coupled to normative pathways, clinicians may experience not only audit pressure and scrutiny, but, in some cases, perceived or actual risk of regulatory or disciplinary consequences when deviating from expected standards. This can further narrow the space for clinical judgement, reinforcing conformity even where variation may better reflect the person's needs, values, or circumstances.

The ethical problem is therefore not the use of evidence or the pursuit of improved outcomes, but the collapse of the boundary between system responsibility and individual authorship. When institutions presume authority over how people ought to live, they move beyond enabling opportunity and into shaping lives directly. This transforms individuals from agents into objects of intervention, and replaces person-authored meaning with system-defined goals. The moral cost of this approach is not only paternalism but fragility. Systems that presume moral certainty struggle to accommodate pluralism, uncertainty, and change. Over time, they weaken trust and erode the very capacities for judgement, responsibility, and self-direction that well-being depends upon.

### 3.2 Ethical abandonment: people as consumers of systems

Ethical abandonment arises when systems retreat from responsibility altogether, framing well-being primarily as a matter of individual choice mediated through markets. This represents the second expression of treating people as objects: objects of transaction whose worth is defined by their capacity to consume services rather than contribute to shared life. Where overreach denies agency by imposing institutional goals, abandonment denies contribution by organizing support entirely around consumption. In both cases, the possibility that people might matter to others beyond taking services is foreclosed.

Here, support is delivered through transactions rather than relationships, and responsibility is formally assigned to individuals without ensuring that meaningful opportunity exists. In this model, people are often described as participants, yet are structurally positioned as consumers or users of systems. Participation is reduced to utilization rather than contribution. Public resources flow toward intermediaries whose incentives prioritize volume, efficiency, or profit, while little attention is paid to whether funded activity cultivates agency, connection, or shared value. The system appears neutral but is structurally indifferent to what is created beyond consumption (28, 31).

When these *laissez-faire* arrangements fail, this is not a failure of personal responsibility. It is a failure of system abdication and of belief in the beneficiaries of the funding. When systems organize support around consumption alone, they implicitly abandon the expectation that people can add to the world around them. Individuals are treated as users of resources rather than as potential contributors to shared life. Support is designed to be taken, exhausted, and replaced, rather than reciprocated or transformed. The result is a one-sided moral economy. Individuals are invited to take without being invited to give; communities are asked to fund support without seeing shared value emerge. Over time, reciprocity erodes, dependence is normalized, and people are reduced to objects of transaction rather than recognized as persons with latent capability.

The risk of consumption-only models is particularly stark for informal carers, who are simultaneously positioned as unpaid contributors to health systems yet rarely recognized as persons with their own needs for support. When support is offered, it often takes the form of respite or services to consume rather than opportunities to contribute or connect beyond their caring roles (32). This exemplifies how systems can simultaneously rely on contribution while designing support that reinforces dependence and consumption. The following Boxes 1 and 2 present illustrative cases. Box 1 illustrates ethical overreach, while Box 2 illustrates ethical abandonment.

Box 1 **Ethical overreach: when evidence shifts from informing choice to directing lives.**

Contemporary medical approaches to well-being provide a useful illustration of the tension between evidence-informed care and personal authorship. Many are increasingly holistic and explicitly oriented toward patient-centered care, autonomy, and beneficence, seeking to address not only physical health but also social, behavioral, and emotional dimensions of well-being. These approaches are typically grounded in strong population-level evidence and aim to support improved outcomes through areas such as diet, movement, social connection, and daily routine (53).

The ethical concern does not lie in the use of evidence, nor in the shift toward more holistic care. It arises when evidence moves from informing choice to directing how people ought to live, without first establishing what matters to the person. In such cases, behavioral pathways are no longer presented as options but become implicitly prioritized. Adherence is treated as success, while deviation may be interpreted as risk, resistance, or failure.

Even where formal choice remains, the authority of evidence can narrow the space for personal authorship, particularly when system-defined goals substitute for a person's own values, priorities, or circumstances. This may reflect an unexamined assumption that evidence-based goals are self-evidently aligned with individual preferences, rather than requiring active elicitation of what matters to the person.

This dynamic is also visible in behavioral policy approaches, including activation strategies and forms of “nudging,” often described as *libertarian paternalism*, where choice is formally preserved but decision environments are structured toward system-preferred outcomes (27, 54). While such approaches may improve uptake and reduce risk at a population level, they risk ethical overreach when behavioral norms become morally directive for individual lives.

The shift is further reinforced where evidence-based practice becomes operationalised as professional expectation. Population-level knowledge, while valuable, can take on a directive force that constrains clinical judgement and reduces tolerance for variation. What begins as evidence-informed practice can become evidence-prescribed practice, where conformity to normative pathways is expected even where these do not align with what matters to the person (55).

Behavioral support remains ethically sound where it serves a person's own goals rather than substituting for them. Approaches such as *shared medical appointments* demonstrate how evidence can be offered without overreach, building capability through peer learning, relational support, and choice (56). In these models, evidence informs action but does not replace the person's authorship over what changes, when, or why.

Box 2 **Ethical abandonment: marketised care and participation without belief.**

Marketised health and social systems risk the opposite failure. Reforms designed to expand choice, control, and participation often assign responsibility to individuals without preserving the conditions required for meaningful opportunity or contribution.

Australia's National Disability Insurance Scheme (NDIS) provides a clear illustration of this (11). At its inception, the NDIS was grounded in an ethically ambitious promise: that people with disability would be supported to pursue their hopes and dreams, to participate socially and economically, and to exercise genuine choice over the lives they wished to lead. Markets were expected to respond creatively, tailoring supports to individual goals and enabling lives of dignity, quality, and contribution.

In practice, this promise was not realized as imagined. Independent review has documented that as supports became marketised, provision stabilized around standardized programs optimized for throughput and risk management rather than growth, imagination, or contribution (57). Participation became attendance; success was measured by utilization, expenditure controls, and compliance oversight, rather than by whether lives expanded in meaning, connection, or authorship. This outcome does not reflect provider or participant failure, but a system-level omission: the absence of an explicit ethical expectation that support should cultivate contribution and shared value.

Without such expectations, markets behaved as markets do. Support was designed to be consumed rather than transformed; choice existed, but within narrow, pre-formed menus. The ethical failure lies not in using markets, but in doing so without belief in people's capacity to matter to others. The result is ethical abandonment: lives are stabilized, but not expanded; supported, but not believed in.

The ethical ambition of schemes such as the NDIS could be strengthened through design and commissioning mechanisms that ask not only how supports align with participant goals, but how plans enable meaningful connection and contribution beyond the boundaries of funded services. This might include simple accreditation requirements that assess whether providers create and maintain opportunities for shared life and contribution, rather than merely facilitating service utilization.

These concepts can be operationalised through simple, experience-based indicators that assess whether people feel valued, respected, and able to contribute to others, alongside measures of whether meaningful opportunities for connection and contribution are available. Where self-report is not appropriate or reliable, indicators of contribution and belonging can be derived from supported reporting, structured observation, and relational measures of engagement and inclusion. Rather than relying solely on activity or service utilization, evaluation can incorporate short self-reported items such as whether individuals feel they matter to others, are needed, or have recently contributed in ways that feel meaningful. At a system level, this allows distinction between participation as attendance and participation as contribution, and provides a pragmatic basis for assessing whether shared life is being sustained.

This pattern is not unique to Australia. England's personalisation reforms under the Care Act 2014, which introduced individual budgets and direct payments to expand choice and control, encountered similar tensions: responsibility was formally assigned to individuals without ensuring that the relational and civic conditions required to exercise choice meaningfully, and to enable contribution, were adequately supported (58).

## 4 Contributory reciprocity: an alternative ethical framework for policy and practice

This paper advances *contributory reciprocity* as an alternative ethical orientation for health and social policy. Contributory reciprocity describes a form of shared life in which individuals are recognized not only as recipients of support, care, or opportunity, but as potential contributors to the lives of others, in ways that are voluntary, plural, and grounded in agency.

If systems fail by treating people as consumers rather than contributors, contributory reciprocity names what must be recovered: the expectation that people can matter to others. This does not impose duties to contribute, nor does it condition support on performance, productivity, or compliance. Instead, it rests on a more demanding ethical claim: that people matter, that what they do can matter to others, and that social systems have a responsibility to preserve the conditions under which such contribution remains possible. This is not about obligation or productivity, but about refusing the assumption that some people can only take and never give — an assumption that, however benevolently enacted, denies human worth and destroys reciprocity.

The ethical failures described in the previous section point to a common absence in contemporary health and social systems: a way to support people that simultaneously believes in their capacity to contribute and invites them to do so, without either prescribing how they should live or abandoning them to market forces. Where systems overreach, they substitute control for trust. Where systems withdraw, they replace reciprocity with transaction. In both cases, shared life erodes.

This ethical orientation draws on several overlapping traditions. It aligns with Aristotelian accounts of flourishing as relational and enacted through shared practices rather than achieved in isolation (6). It resonates with ‘civic republican' concerns about non-domination, in rejecting systems that remove agency by substituting control for trust (33). It sits alongside capability approaches, which emphasize what people are able to be and do, while often remaining ethically agnostic about contribution to others (12, 13). It also reflects insights from relational ethics, which locate moral worth not solely in autonomy or independence, but in the capacity to stand in meaningful relation to others (34, 35).

Contributory reciprocity differs from participation-based models that frame inclusion primarily as attendance within predesigned activities. Participation, while often well intentioned, allows people to be present without being recognized as authors of shared life. Contribution, by contrast, implies that a person's presence, effort, or expression can shape the social world, however modestly. Contribution need not be economic, instrumental, or formally productive. It may take the form of joy, care, attention, creativity, encouragement, hospitality, reliability, cultural expression, or simply showing up in ways that strengthen belonging.

Contributory reciprocity shares intellectual terrain with several established approaches while remaining distinct in important ways. These distinctions are summarized in Table 1. It aligns with an Asset-Based Community Development (ABCD) approach in emphasizing community capacity and contribution over deficit-focused service delivery (15), extending this by situating contribution within a broader ethical framework of responsibility-partitioning and civic expectation. It resonates with co-production approaches that position citizens as producers rather than passive consumers of services (14, 36), but differs in refusing to instrumentalise contribution as a means of improving service efficiency or reducing costs. Where co-production often focuses on individuals contributing to their own services, contributory reciprocity emphasizes contribution to shared life beyond individual benefit. Most critically, contributory reciprocity must be distinguished from neoliberal activation or welfare conditionality. The latter makes support conditional on contribution, treats contribution as obligation, and defines it narrowly as labor market participation (37). Contributory reciprocity, by contrast, does not condition support on contribution. It treats contribution as voluntary and plural in form, and recognizes non-economic forms of value that cannot and should not be measured, monetised, or mandated. The difference is between coercion and invitation, between obligation and possibility, and between instrumental participation and intrinsic worth.

**Table 1:** Contributory reciprocityin relation to adjacent frameworks.

Framework	Core focus	Relationship to contribution	Key distinction from contributory reciprocity
Capability approach [Sen (12); Nussbaum (13)]	What individuals are able to be and do	Contribution is not a primary concern; focus is on individual functioning and freedom	Contributory reciprocity explicitly emphasizes contribution to others, not only individual capability
Co-production [Ostrom (14); Pestoff (36)]	Citizens as producers of their own services	Contribution is instrumental: it improves service efficiency and reduces costs	Contributory reciprocity refuses to instrumentalise contribution; emphasizes contribution to shared life beyond individual benefit
Asset-based community development [Kretzmann and McKnight (15)]	Community capacity and strengths over deficit	Contribution is valued but situated within community asset-mapping	Contributory reciprocity situates contribution within a responsibility-partitioned ethical framework that also addresses what systems owe individuals
Welfare conditionality [Dwyer (37)]	Compliance with obligations as condition of support	Contribution is mandated, narrowly defined as labor market participation	Contributory reciprocity treats contribution as voluntary, plural, and unconditional on support

These non-economic contributions can be understood as *social sweat equity:* the everyday investment of time, presence, care, and effort through which shared life is sustained. This notion reflects long-standing civic understandings of reciprocity, in which the maintenance of common life depends not on constant productivity, but on people contributing what they can, when they can, in forms shaped by circumstance (19, 38). Social sweat equity is not a substitute for structural justice or material support, but a moral dimension of shared life that cannot be supplied by systems alone and must therefore be invited.

### 4.1 Why contribution matters: meaning, belonging, and social fabric

Contributory reciprocity rests on a simple but often neglected moral insight: that to be useful to others is itself a source of meaning, and that shared life depends on people having ways to add value beyond consumption or compliance. Contribution matters not because it can be demanded, measured, or optimized, but because it is one of the primary ways through which people come to matter to others, and through which social life becomes meaningful rather than merely busy.

Contribution need not be dramatic, enduring, or identity-defining. It often takes the form of small, ordinary acts whose value lies precisely in their ordinariness: showing up, helping, affirming, noticing, listening, maintaining, encouraging, or taking responsibility for something that others rely upon. Such acts generate what might be described as small meaning — not a grand narrative of purpose, but the practical experience of feeling of use or importance to others. This form of meaning is relational rather than introspective. It does not arise primarily from self-expression or internal coherence, but from adding value to others in ways that are recognized, reciprocated, or relied upon. Through contribution, people come to matter concretely not abstractly.

Contemporary well-being systems operate on a fundamental misconception: that belonging can be created by granting inclusion and by ensuring people can access activities, attend programs, or participate in spaces. This gets the causal sequence backwards. Belonging is not a precondition that systems provide before people engage; it emerges through contribution itself. People come to belong not when they are permitted to show up, but when they discover that their actions make a difference to shared life, when what they do is noticed, relied upon, or valued by others. Inclusion may create attendance; only contribution creates belonging.

It is through contribution, particularly across diverse groups and settings, that social fabric itself is constructed (19). Putnam's concept of bridging social capital describes precisely this: the trust, reciprocity, and mutual recognition generated when people contribute alongside others who differ from them in circumstance, background, or capability. This social wealth cannot be delivered through programs that segregate people into homogeneous groups, nor through services that position everyone as recipients. It emerges only where people act as contributors to shared endeavors. Systems that never invite contribution therefore actively prevent the formation of the social capital on which communities, democracies, and shared life depend. A society organized entirely around service consumption is not only atomising for individuals, but structurally incapable of generating the trust, reciprocity, and mutual recognition that hold diverse populations together.

Thus, contributory reciprocity is essential for the very survival of systems and shared life. Over time, small acts of contribution accumulate. Repeated usefulness generates familiarity, trust, informal obligation, and mutual expectation — the basic elements of social capital and social wealth. These are not outcomes that systems can directly deliver. They emerge only where people are able to act as contributors rather than solely as recipients or consumers of support.

### 4.2 Three enabling conditions for contributory reciprocity

For contributory reciprocity to be more than rhetorical, systems must meet three specific enabling conditions: preserving real opportunity, protecting a civic canvas where shared life can occur, and extending genuine invitation. These conditions are necessary, but not sufficient. The irreducible element is contribution itself — the act, however modest, through which a person's presence, effort, attention, or care becomes useful to someone else. Systems cannot manufacture contribution, but they can preserve the conditions under which it becomes possible.

**First: Preserving real opportunity**. Opportunity must be materially, socially, and culturally accessible, not merely programmatic or theoretical. People cannot contribute if there is nowhere to do so, no pathway for entry, no tolerance for experimentation and failure, or no margin for imperfect participation. Systems that fully enclose social life within markets or tightly specified programs risk converting opportunity into commodity and contribution into consumption.

**Second: Protecting the civic canvas**. Civic canvases are the shared physical, social, and institutional spaces within which collective life can be shaped, including community halls, libraries, parks, beaches, workshops, cultural venues, places of worship, informal clubs, and other commons that remain open to use without excessive cost, permission, or professional mediation. A civic canvas is not simply infrastructure, but a permission structure: it signals that people are able to gather, organize, create, and contribute without needing to justify their presence through productivity or purchase. In this sense, civic canvases overlap with what Klinenberg (39) describes as “social infrastructure”: the physical and relational environments that shape whether connection, participation, resilience, and shared life become possible.

Civic canvases are not limited to physical or recreational spaces. They also include cultural, spiritual, and symbolic domains through which people make sense of their lives and locate themselves in relation to others. Rituals, shared narratives, artistic practices, faith communities, and moral traditions function as canvases in the same ethical sense: they provide spaces where contribution is possible without prior permission, where presence carries meaning, and where people can matter beyond market or clinical rules. When these domains are marginalized or professionalized, shared life thins even if physical spaces remain intact.

When civic canvases are absent, over-regulated, or enclosed by market mechanisms — through fees, platforms, intermediaries, or professionalized programs — contributory reciprocity becomes difficult or impossible. By contrast, cities that have deliberately maintained accessible civic infrastructure, such as Barcelona's pedestrianized superblocks, demonstrate that preserving shared space as a non-market resource is a viable policy choice rather than an incidental outcome (40).

In some communities, particularly rural, disadvantaged, highly marketised, or socially fragmented settings, the civic canvas may be weak, exclusionary, or largely absent. Under such conditions, contributory reciprocity cannot rely solely on navigation into existing opportunities. Instead, it may require active cultivation of the civic conditions within which contribution becomes possible: protecting informal spaces, enabling low-barrier community infrastructure, supporting micro-organizing, reducing exclusionary practices, strengthening local capability, and helping latent opportunities emerge over time.

Importantly, fragmented civic environments should not be misinterpreted as evidence that people lack willingness, capability, or desire to contribute to others. Contribution can and does emerge informally through neighborliness, care, friendship, family life, and everyday reciprocity even under constrained conditions. However, where shared civic spaces and accessible opportunities for participation are weak or inaccessible, systems may make contribution, connection, and shared life unnecessarily difficult to sustain beyond immediate personal networks.

**Third: Extending genuine invitation**. Opportunity and space alone are insufficient. Many opportunities remain unused not because people lack capacity or interest, but because they lack familiarity, confidence, or a sense of belonging. Invitation makes possibility visible and communicates that a person's presence would be welcome. It signals permission, rather than prescription.

Invitation is ethically significant because it signals not only access, but expectation: the expectation that a person is capable of contributing something, however modest or intermittent. To invite contribution is to treat people as agents whose presence can add value to shared life. This reflects a minimal civic assumption that people, when able, can add something to shared life. Without this expectation, systems risk withdrawing belief in people's capacity and leaving opportunities unrealised in practice, regardless of how much provision exists in principle.

This expectation must remain non-coercive. Contribution is voluntary, plural, and uneven across lives and circumstances. Illness, disability, age, trauma, and context shape how and when people contribute. It is rarely constant, symmetrical, or uniform. What matters ethically is not the quantity or visibility of contribution, but the recognition of capacity and the openness of invitation. A system that never asks how someone might contribute has already answered that question on their behalf.

Table 2 maps observable indicators against the enabling conditions of contributory reciprocity, alongside contribution as an emergent outcome.

**Table 2:** Observable indicators of contributory reciprocity in system design and practice.

Condition	Indicator	What it assesses
Opportunity	Use of opt-in invitation approaches rather than default enrolment or passive assignment	Whether systems presume capability rather than incapacity
	Evaluation frameworks separately measure intervention reach and individual uptake	Whether systems analytically distinguish between invitation and response
	Non-uptake is not recorded as program failure or individual deficit	Whether systems respect agency at the boundary of responsibility
Civic canvas	Availability of accessible, low-cost, non-programmatic community spaces (e.g., “third places”)	Whether shared spaces for contribution exist outside formal services
	Support for informal, community-led, or non-professionalized forms of activity	Whether contribution can emerge without mediation or permission
Invitation	Presence of link worker or navigator roles with explicit invitation function	Whether systems actively communicate that a person's presence would be welcome and valued
	Policy and commissioning language distinguishes contribution from participation	Whether systems move beyond attendance-based frameworks
Contribution	People report feeling recognized as capable of adding value to others	Whether people feel recognized as capable of adding value to others
	Funded activities include roles for participants beyond passive receipt	Whether contribution is structurally enabled
	Well-being plans include questions about how a person might contribute	Whether systems explicitly invite contribution at the individual level

When John F. Kennedy (41) urged citizens to “ask not what your country can do for you,” he spoke in a social and technological context that presumed participation in shared civic life as the default. That presumption no longer holds. Contemporary societies increasingly organize daily life around frictionless consumption, with food delivered to the door, entertainment to the bedroom, services mediated by platforms, and artificial intelligence available instantly in the hand. In this context, contribution cannot be assumed. It must be structurally invited.

The ethical task of systems is therefore not to secure contribution as a performance outcome, but to preserve the conditions under which contribution remains possible and meaningful. Where those conditions are sustained, meaning, belonging, and social wealth may grow. Where they are absent, shared life predictably thins, regardless of how much support is delivered.

These ethical stakes are no longer abstract. As automation and artificial intelligence reduce the centrality of paid labor, and as some societies experiment with income decoupled from contribution, the risk of a consumption-first social model intensifies. Where expectation, reciprocity, and shared purpose are already thin, such shifts may accelerate lives organized around use rather than belonging, deepening disconnection regardless of how much support is delivered.

This ethical stance occupies a demanding middle ground. It affirms expectation without coercion, support without prescription, and belief without naivety. Shared life cannot be manufactured, but it can be sustained. Where belief is maintained, contribution remains possible.

## 5 Social prescribing as illustrative policy mechanism

Contemporary health and social systems are largely organized around the identification and remediation of illness, injury, disability, pathology, risk, and deficit. In itself, this is not ethically problematic. Such systems are often motivated by beneficence and the reduction of suffering. However, when people are encountered primarily through deficit-based frameworks, systems may unintentionally narrow personhood to need, vulnerability, diagnosis, or impairment (42, 43). In doing so, they risk reproducing the forms of ethical overreach and ethical abandonment described earlier: either intervening excessively into people's lives while displacing agency, or reducing people to passive recipients of care whose capacity to matter, contribute, participate, or shape shared life is no longer meaningfully recognized.

The ethical framework developed here does not depend on social prescribing specifically. Rather, social prescribing is presented as one illustrative example of how systems might operationally preserve opportunities for participation, recognition, belonging, contribution, and shared life alongside care and support. Social prescribing is useful insofar as it illustrates how systems can bridge the gap between opportunity and agency without collapsing into either overreach or abandonment. When practiced with restraint, social prescribing demonstrates how preserved opportunity, accessible civic and community spaces (the civic canvas), and relational invitation can be held together in a way that supports shared life while respecting pluralism and responsibility.

In policy and practice, social prescribing is commonly defined in functional terms. NHS England describes it as a means of connecting people to non-clinical support within their community to improve health and well-being (44). Early academic accounts similarly emphasized linkage, navigation, and referral to community-based resources (45, 46). More recent consensus work, including a Delphi-derived definition, has reinforced this focus on connection, personalisation, co-production and community engagement (47). International guidance, including the World Health Organization's toolkit on implementing social prescribing, similarly frames the practice in functional terms as a means of connecting individuals to community resources (48). These definitions are intentionally broad and operational. They describe what social prescribing does but leave open the more ethically significant question of why it matters (49).

An integrative review of Australian link worker programs confirms this functional orientation, identifying diverse outcome domains including mental health, well-being, social connection, and health service utilization (50). Empirical evidence from the UK and Australia suggests that social prescribing is associated with improvements in mental well-being, reduced social isolation, and increased community participation, although heterogeneity across program models makes direct comparison difficult (46, 50). These findings are consistent with the argument developed here: that invitational approaches support agency and connection without prescribing outcomes.

What remains striking across these definitions and evaluations is that quality of life is almost always treated as an outcome to be improved (50), rather than as a concept to be examined. Measures of well-being, mental health, and social connection are frequently used as proxies for quality of life, yet rarely interrogate what kind of life is being supported, or whether systems are strengthening the conditions for shared life rather than simply mitigating distress. This leaves unanswered the ethical question of whether practices designed to “improve” quality of life also preserve agency, authorship, and the capacity to contribute within shared life. Where systems measure only whether people feel better, without asking whether people retain the capacity to shape their own lives or matter to others, they risk mistaking reduced distress for ethical success rather than asking whether shared life has been meaningfully preserved.

From an ethical perspective, social prescribing is best understood not as the delivery of activities or experiences, but as an invitational practice that emphasizes relational accompaniment, autonomy, and preserving personal authorship rather than directing predetermined behavioral outcomes (51). Its function is to help people see that there is a canvas on which life might be lived differently and that they are permitted to engage with it rather than determine what people should do. It does not paint the picture on a person's behalf. It points to the canvas, names the colors that are available, and offers brief accompaniment while the person decides whether, how, and what they wish to create. The work of meaning, motivation, and contribution remains with the individual. The role of the system is preparatory rather than productive.

This distinction matters because social prescribing is easily misinterpreted as a form of behavioral prescription. When it operates by directing individuals toward predefined activities on the basis of assumed benefit, it reproduces the ethical overreach described earlier. When it functions primarily through the provision of commodified experiences, treating social connection, culture, or participation as “doses” to be dispensed, it risks reinforcing the logic of consumption.

Seen through the canvas metaphor, the limits of social prescribing become clear. It cannot create meaning on behalf of a person, just as it cannot guarantee that a painting will be made. Nor should it attempt to decide what the finished picture ought to look like. Its role is to preserve possibility, not to dictate outcome. Where this boundary is crossed, social prescribing ceases to be invitational and becomes another form of managed participation.

At the same time, social prescribing is not ethically neutral. Invitation carries an implicit recognition that a person's presence and effort could matter and that the canvas is not merely there to be admired, but to be used. In this sense, social prescribing quietly resists the reduction of individuals to consumers of support. It affirms that contribution, however modest or informal, remains possible. Whether someone chooses to paint is ultimately their decision, but being shown that painting is allowed, and that others may value what they create, is ethically significant. This emphasis on contribution and relational value aligns with equity-informed approaches to health and well-being (52) and reflects community-centered accounts of social prescribing.

Social prescribing therefore occupies a narrow but important space between system responsibility and individual agency. It does not expand the system's authority to shape lives, nor does it withdraw into *laissez-faire* indifference. Instead, it operates as a bridge: pointing toward preserved opportunity, lowering the threshold for entry, and then stepping aside. When practiced in this way, social prescribing exemplifies an ethical posture grounded in dignity, pluralism, and contributory reciprocity. Table 3 summarises the shift from beneficence-focused to reciprocity-focused system questions.

**Table 3:** Beneficence-focused vs. reciprocity-focused system questions.

Questions systems ask	Questions systems avoid
Did the person feel better?	Did the person matter to anyone?
Did their distress reduce?	Did anyone rely on them?
Was the activity delivered?	Did it create reciprocal meaning?
Was engagement logged?	Did contribution become possible?
Was access provided?	Would anyone notice if they were absent?

Table 4 provides a comparison of what invitational and transactional social prescribing look like in practice. When this posture of invitation is lost or becomes prescription, or when the canvas is replaced by pre-packaged experiences, the practice forfeits its distinctiveness. What remains is not social prescribing in the sense articulated here, but another variant of consumption-oriented support. The value of social prescribing lies not in the activities it connects people to, but in its refusal to decide in advance what a *good life* should look like.

**Table 4:** Distinguishing Invitational from transactional social prescribing.

Dimension	Invitational approach	Transactional approach
**Orientation to opportunity**	Orients people toward opportunities in civic, cultural, and community life	Delivers activities or experiences as predefined interventions
**Treatment of connection**	Treats social connection and participation as relational possibilities	Treats social connection and participation as commodities or “doses”
**Locus of authorship**	Preserves authorship by allowing individuals to decide whether, how, and when to engage	Substitutes system judgement for personal authorship through assumed benefit
**Temporal boundary**	Operates as time-limited accompaniment, stepping back once access becomes usable	Risks becoming ongoing provision or managed participation
**Recognition of people**	Affirms people as potential contributors to shared life	Positions people primarily as recipients or consumers of support
**Response to non-uptake**	Accepts plural outcomes, including refusal or disengagement	Frames non-uptake as failure, resistance, or poor compliance
**Ethical stance**	Resists paternalism without abandoning responsibility	Reproduces either behavioral control or market-driven indifference
**Ethical distinction**	Invitational social prescribing preserves dignity by recognizing that people matter and that what they do could matter to others.	Transactional approaches may improve access or enjoyment, but they risk collapsing invitation into consumption, and contribution into attendance.

## 6 Actionable recommendations for policy, commissioning, and system design

The implications of contributory reciprocity are not primarily technical, but structural. They require systems to shift from delivering services and activities focused on throughput and short-term outputs toward preserving the conditions under which contribution, connection, and shared life can emerge. Across domains, a consistent pattern follows: systems must distinguish clearly between what they are responsible for providing, including access, capability, opportunity, and invitation, and what individuals retain as authors of their own lives, including meaning-making, choice, and whether and how to engage.

This shift extends beyond supporting individual choice. It requires systems to recognize people not only as recipients of care, but as potential contributors to shared life, whose presence, effort, and participation can matter to others. Contribution is not an obligation to be enforced, but a possibility to be preserved and invited. Systems therefore have a responsibility to make visible the ways in which people can add value to their communities, to create conditions where this is possible, and to signal that such contribution is welcomed and meaningful. Without this invitation, support risks being organized entirely around consumption, leaving individuals disconnected from the reciprocal relationships through which belonging and social value are formed.

These implications carry directly into how policy is written, how services are designed and commissioned, how outcomes are measured, and how professional practice is understood. It requires moving beyond models that prioritize throughput, compliance, or activity delivery, toward approaches that center relational work, neighborhood and community infrastructure, and the civic contexts in which contribution and belonging can occur.

At its core, contributory reciprocity must be made operational within systems. This includes embedding practices that actively elicit what matters to the person, while also exploring how individuals may engage with, shape, or contribute to the world around them. In this sense, informed consent is not merely procedural agreement, but a process through which individuals understand, interpret, and endorse the actions affecting their lives, and locate those actions within their own values, priorities, and relationships. Evidence remains essential, but it must be held in balance with agency, clinical judgement, and the person's own goals.

It also requires strengthening relational and neighborhood-oriented approaches to health and well-being. Contribution does not occur in isolation or within services alone, but within shared civic, social, and cultural contexts. Systems must therefore invest in the relational workforce, local networks, and civic infrastructure that make it possible for people to connect, participate, and contribute beyond formal care settings. These conditions form the civic fabric through which social capital is created and sustained.

The following recommendations translate contributory reciprocity from ethical orientation into operational practice. They are organized by domain (design, commissioning, evaluation, policy, and implementation) and share a common logic: preserving contribution as possibility rather than obligation, maintaining the boundary between system responsibility and individual authorship, and guarding against drift toward models organized solely around consumption.

Taken together, these domains form a coherent system model for operationalising contributory reciprocity: (1) systems must design for contribution, (2) fund the conditions that sustain it, (3) measure outcomes without collapsing agency, (4) embed these principles in policy, and (5) protect them from drift in implementation (see Table 5).

**Table 5:** Operationalising contributory reciprocity across system domains.

Domain	System responsibility	What is preserved for the individual	Key risk if collapsed
**Design**	Structure opportunity, access, and invitation into shared life	Choice of how, when, and whether to engage and contribute	Managed participation replaces shared life
**Commissioning**	Fund relational work, access pathways, and civic infrastructure	Autonomy in participation and contribution	Consumption is incentivised over contribution
**Evaluation**	Assess opportunity, invitation, and barrier removal	Agency in response, including non-participation	Non-engagement misclassified as failure
**Policy**	Define enabling conditions and responsibility boundaries	Authorship, plural values, and meaning-making	Paternalism or abandonment
**Implementation**	Maintain fidelity to invitation, relational practice, and flexibility	Independent participation and informal contribution	Drift toward prescription, dependency, or commodification

These recommendations apply across disability, aged care, mental health, and community health systems. While illustrated through social prescribing, they are relevant to any system seeking to balance support with agency, and inclusion with contribution.

### 6.1 Recommendations for system design - *design for contribution*

From a design perspective, the central task is to structure opportunity, invitation, and the conditions under which people can contribute to shared life, rather than prescribing activity. Ethical design asks not what people should do, but whether they can realistically see, approach, and enter shared civic and community contexts without excessive difficulty, risk, or gatekeeping, and whether they are recognized as capable of adding value within them.

Overly specified systems risk replacing shared life with managed participation, narrowing the space in which contribution can occur. Access alone is insufficient if people are not invited, supported, and recognized as potential contributors whose presence and participation can matter to others.

Systems should implement the following design principles:

#### Recommendation 6.1.1: Design for opportunity and contribution, not activity delivery

Prioritize creating conditions under which contribution becomes possible rather than specifying what forms contribution should take. This includes ensuring material, physical, social, and temporal access to shared spaces, alongside opportunities for individuals to participate in ways that are meaningful to them and valuable to others.

#### Recommendation 6.1.2: Enable low-threshold, plural, and flexible entry and contribution

Allow people to try, withdraw, and re-enter civic and community spaces without penalty, professional gatekeeping, or permanent records of non-compliance. Recognize that contribution takes many forms, is often informal, and rarely follows linear trajectories.

#### Recommendation 6.1.3: Embed invitation as a core design function

Ensure that systems actively communicate that a person's presence would be welcome and that they are capable of contributing in some way. This includes making opportunities visible and using relational roles to extend invitation, without requiring prior readiness, confidence, or qualification.

#### Recommendation 6.1.4: Practice ethical restraint in system design

Leave room for uncertainty, improvisation, and ordinary social life. Over-designed systems risk crowding out informal and emergent forms of contribution by replacing shared space with managed participation or prescribed pathways.

**Policy Example:** Current NDIS participation goals risk conflating access to activities with meaningful contribution. A contributory reciprocity approach distinguishes between enabling access to community spaces (system responsibility) and the form of participation or contribution chosen (individual responsibility), supporting the former without mandating the latter. It would also require systems to make visible and accessible pathways through which individuals can contribute, whether through informal roles, peer support, shared activities, or everyday participation, and to signal that such contributions are welcomed and valued. In this way, systems move beyond facilitating attendance toward creating the conditions in which people can matter to others.

### 6.2 Recommendations for commissioning and funding - *fund the conditions for contribution*

Commissioning decisions signal what systems value. Where funding is tied primarily to attendance, throughput, or activity completion, systems default toward consumption rather than contribution. Providers are incentivised to deliver occasions of service rather than to cultivate capability, confidence, connection, or the conditions under which people can contribute to shared life.

A contributory reciprocity approach requires commissioning frameworks to distinguish clearly between what systems are responsible for funding, including access, opportunity, and relational support, and what individuals retain as authors of their own engagement, including whether and how they participate or contribute.

Commissioners should adopt the following principles:

#### Recommendation 6.2.1: Fund relational work as core infrastructure, not administrative overhead

Commission and adequately resource roles that enable connection, invitation, and bridge-building, including link workers, community development practitioners, and peer roles. These functions should be recognized as central to system performance rather than ancillary to service delivery.

#### Recommendation 6.2.2: Commission for capability and contribution, not sustained consumption

Fund models that build confidence, familiarity, and capacity to engage in shared life, rather than those that require ongoing participation in funded activities. Commissioning should support pathways into contribution, not dependence on service provision.

#### Recommendation 6.2.3: Separate funding for opportunity from funding for activity

Distinguish between investment in enabling conditions, such as civic spaces, community infrastructure, and access pathways, and funding for specific programs or activities. This allows systems to remain accountable for creating opportunity without prescribing how individuals should engage.

#### Recommendation 6.2.4: Protect the civic canvas from full market enclosure

Maintain public investment in shared civic infrastructure, including community spaces, libraries, parks, and local organizations, that enable contribution without transaction. Avoid funding models that convert all forms of participation into fee-for-service provision or professionally mediated activity.

#### Recommendation 6.2.5: Design contracts that tolerate variation and exercise ethical patience

Allow natural variation in uptake, timing, and forms of engagement. Avoid rigid participation quotas or performance metrics that penalize programs serving complex populations or create pressure to drive attendance. Value may be created indirectly, through restored confidence, informal participation, or renewed willingness to engage, without immediate or easily measurable outputs.

**Policy Example:** Australian Primary Health Networks commissioning social prescribing could distinguish between link worker time, as relational work that enables connection, agency, and invitation, and activity provision, as the opportunities people may choose to engage with, funding both appropriately while recognizing their different roles and timeframes. This approach would also support pathways through which individuals can move from connection to participation and, where desired, into forms of contribution within their communities, without making such progression a requirement.

**Policy Example:** UK Integrated Care Systems could include explicit commissioning for the preservation of civic infrastructure alongside activity-based contracts, recognizing that shared spaces require sustained investment even when utilization fluctuates. These settings form the conditions within which contribution can occur, enabling informal participation, social roles, and everyday acts of contribution that are not easily specified or contracted but are central to shared life.

### 6.3 Recommendations for evaluation and accountability - *measure without collapsing agency*

Evaluation is where ethical intent is most easily undermined. Frameworks that equate success with uptake, attendance, or compliance risk misclassifying agency as failure and participation as an end in itself. Within a contributory reciprocity framework, this represents a category error: systems are responsible for creating opportunity and extending genuine invitation, while individuals retain responsibility for whether and how they choose to engage.

Evaluation must therefore distinguish clearly between what systems provide and what individuals choose. Without this distinction, responsibility collapses, and both system performance and individual agency become obscured.

Evaluation frameworks should implement the following principles:

#### Recommendation 6.3.1: Distinguish system provision from individual response

Assess separately whether opportunity has been made real and accessible, whether genuine invitation has been extended and communicated, and whether barriers have been removed, as measures of system performance. These should not be conflated with whether individuals choose to engage or what outcomes they achieve.

#### Recommendation 6.3.2: Do not treat non-engagement as failure

Non-engagement following genuine invitation should not be recorded as program failure or individual deficit. Declining, delaying, or withdrawing from participation may reflect preference, timing, readiness, or competing priorities, and should be recognized as an expression of agency rather than a lack of compliance.

#### Recommendation 6.3.3: Incorporate qualitative indicators of agency and recognition

Include measures that assess whether individuals understand their options, feel recognized as capable, and perceive themselves as potential contributors to shared life. These indicators capture whether systems are supporting authorship and possibility, not only whether activities are attended.

#### Recommendation 6.3.4: Maintain asymmetrical accountability

Systems remain accountable for preserving opportunity, removing barriers, and extending invitation. Individuals remain accountable for how they choose to respond. Collapsing these responsibilities into a single performance metric obscures agency on both sides and incentivises pressure toward participation.

#### Recommendation 6.3.5: Distinguish reach from uptake

Differentiate between whether people were made aware of and genuinely invited to opportunities (reach) and whether they chose to engage (uptake). Low uptake should not be interpreted as straightforward program failure without first examining whether opportunity and invitation were meaningful and accessible.

**Policy Example:** International evaluation frameworks addressing social connection, including those emerging from World Health Organization initiatives, could be strengthened by distinguishing between intervention reach and individual uptake, rather than treating low participation as a direct indicator of program ineffectiveness. They could also assess whether meaningful invitation has been extended and whether individuals feel recognized as capable of contributing to shared life, ensuring that evaluation captures not only participation, but the conditions that make contribution possible.

**Policy Example:** Within the NDIS, evaluation of participation could be refined by distinguishing between whether systems have created genuine opportunities for contribution and whether individuals have chosen to take them up. Treating non-uptake as an outcome deficit risks collapsing voluntary participation into compliance rather than recognizing agency. A contributory reciprocity approach would also assess whether individuals were supported to identify what matters to them and whether opportunities for contribution were visible, accessible, and aligned with those priorities.

### 6.4 Recommendations for policy development – *embed in policy*

At the policy level, contributory reciprocity requires embedding a clear distinction between system responsibility and individual authorship within legislation, strategy, and national frameworks governing health, social care, disability, and aging. Without this, policies risk drifting toward either paternalism, where systems prescribe how people ought to live, or abandonment, where responsibility is devolved without the conditions required to exercise it.

Policy must therefore do more than expand access or participation. It must define the conditions under which people can both shape their own lives and be recognized as contributors to shared civic and community life.

Policymakers should adopt the following principles:

#### Recommendation 6.4.1: Distinguish participation from contribution in policy language

Policy objectives that emphasize participation should clarify whether the goal is presence in activities or recognition as contributors to shared life. These are not equivalent and require different policy architectures. Policies should explicitly support contribution as a possibility, without mandating it as an obligation.

#### Recommendation 6.4.2: Embed responsibility partitioning within rights-based frameworks

Legislation and strategy should clearly define what systems are responsible for providing, including access, capability, opportunity, and invitation, and what individuals retain, including authorship, meaning-making, and choice of engagement. This helps avoid both overreach and abdication of responsibility.

#### Recommendation 6.4.3: Require elicitation of what matters to the person as a core policy expectation

Policies should mandate that services actively identify and respond to what matters to individuals, rather than assuming alignment with standardized goals or population-level evidence. This includes recognizing that goals, priorities, and definitions of well-being are plural and context-dependent.

#### Recommendation 6.4.4: Balance evidence with agency in policy design and guidance

Policy should support the use of evidence to inform care and decision-making, while explicitly protecting the role of individual preference, clinical judgement, and contextual interpretation. Evidence should guide options, not prescribe lives.

#### Recommendation 6.4.5: Preserve and invest in the civic canvas as a public good

Policy frameworks should include explicit commitments to maintaining shared civic infrastructure, including public spaces, community venues, local networks, and informal “third places,” that enable contribution, connection, and belonging. These should be recognized as foundational components of well-being, not optional adjuncts to service delivery.

#### Recommendation 6.4.6: Avoid prescribing substantive life goals within policy frameworks

Policy should specify enabling conditions, including access, opportunity, and capability, without defining what constitutes a good life or desirable behavior for diverse populations. This preserves pluralism and protects individual authorship.

**Policy Example:** National social prescribing frameworks could explicitly adopt contributory reciprocity principles by stating that systems are responsible for ensuring genuine access to community life, while individuals retain responsibility for whether and how they engage. This could be extended by requiring systems to make visible and accessible opportunities for contribution, and to recognize and value the diverse ways in which individuals contribute to shared life, without mandating contribution as an outcome.

**Policy Example:** Australia's Aged Care Act reforms could distinguish between enabling older people to access and connect with community, as a system responsibility, and supporting, but not mandating, contribution, ensuring that engagement remains voluntary and aligned with individual priorities. A contributory reciprocity approach would also embed expectations that services identify what matters to individuals and create opportunities for contribution that reflect these preferences, recognizing older people not only as recipients of care, but as contributors to shared community life.

### 6.5 Preventing ethical drift in implementation – *protect from drift*

The application of contributory reciprocity in practice is vulnerable to ethical drift as systems scale, professionalize, or become subject to performance pressures and market incentives. Approaches that begin as relational, invitational, and oriented toward contribution can gradually shift toward prescription, standardization, or commodification, particularly where accountability frameworks prioritize measurable outputs over relational and social value.

Preventing this drift requires ongoing attention to how systems are enacted in practice, not only how they are designed in principle. Without this, the language of contribution, choice, and person-centered care can be adopted while underlying practices continue to prioritize compliance, throughput, or behavioral conformity.

Systems should implement the following safeguards:

#### Recommendation 6.5.1: Monitor for drift from invitation to prescription

Identify when flexible, person-led pathways are being replaced by standardized menus of activities, or when options presented as choice become implicitly expected or directed. This includes recognizing when social prescribing or similar approaches begin to resemble structured intervention pathways rather than open invitations into shared life.

#### Recommendation 6.5.2: Resist performance pressures that incentivise participation as an outcome

Push back against funding, reporting, or governance expectations that equate success with attendance, uptake, or activity completion. These pressures can lead to subtle or overt encouragement of participation, undermining voluntary engagement and distorting the meaning of contribution.

#### Recommendation 6.5.3: Maintain time limits on intermediary roles and avoid dependency drift

Ensure that roles such as link workers, navigators, or coordinators remain focused on enabling entry, connection, and confidence-building, rather than evolving into sustained case management. Ongoing dependence on intermediary roles can displace opportunities for independent participation and contribution.

#### Recommendation 6.5.4: Protect informal and community-led forms of contribution

Recognize and preserve forms of participation that occur outside formal programs, including informal helping, social roles, and everyday contributions within communities. Avoid over-professionalizing or commodifying these activities in ways that narrow how contribution is recognized.

#### Recommendation 6.5.5: Embed ongoing ethical reflexivity in governance and practice

Establish processes that regularly examine whether systems are enabling contribution or managing consumption, and whether they are supporting agency or creating dependency. This includes asking: *Are we helping people connect to shared life, or are we organizing participation on their behalf? Are we preserving authorship, or subtly replacing it?*

**Policy Example:** Regular ethical audits of social prescribing or community-based programs could assess whether practitioners are being pressured to maintain ongoing relationships or drive participation, rather than supporting transition into independent engagement and contribution. Such audits could also examine whether practice remains oriented toward invitation and connection, or has drifted toward implicit expectation, compliance, or dependency.

**Policy Example:** Commissioning and reporting frameworks could be reviewed to identify whether performance metrics, such as attendance targets or completion rates, are creating implicit pressure to drive participation. Where such pressures exist, programs may drift from invitation toward expectation, subtly reshaping voluntary engagement into managed compliance. Adjusting these settings to prioritize opportunity, invitation, and independent transition can help preserve the integrity of contributory reciprocity in practice.

### 6.6 Implementation considerations – *consider realities and trade-offs*

These recommendations are offered as ethical repositioning rather than technical fixes, and their implementation involves real constraints and trade-offs that warrant acknowledgment. They are most applicable where some degree of civic infrastructure, relational workforce capacity, and commissioning flexibility already exists; in contexts where these have been significantly eroded or were never established, the framework identifies what needs to be built rather than what can be immediately implemented. Reorienting commissioning and evaluation toward relational work, opportunity provision, and ethical patience requires accepting reduced short-term measurability, which creates genuine tension with existing accountability frameworks, performance reporting requirements, and funder expectations that prioritize throughput and demonstrable outcomes. These recommendations do not propose a low-cost alternative to service delivery; sustaining relational workforce roles, protecting civic infrastructure, and commissioning for contribution rather than consumption all require deliberate and sustained investment, with returns that may be diffuse, delayed, and difficult to attribute to specific interventions. The resource and governance implications of this reorientation are substantial, and their practical management will depend on local context, political will, and the courage of commissioners and policymakers to fund what is difficult to measure. These challenges are discussed further in the limitations section.

## 7 Discussion: implications for health and social policy

The ethical challenge facing contemporary health and social systems is not only how to provide support, but how to sustain shared life under conditions of social fragmentation, technological change, and declining civic participation. As paid labor becomes less central to economic survival for some, and as automation and artificial intelligence reshape work, welfare, and social organization, the question of contribution can no longer be deferred. The issue is no longer simply how income is secured, but how meaning, reciprocity, and mutual need are preserved in societies where traditional forms of contribution are increasingly disrupted (2).

Three forward-looking implications emerge from this analysis.

**First, the trajectory of welfare systems matters ethically, not only economically**. As societies experiment with expanded welfare provision, universal basic income, and increasingly technologically mediated life, the risk of a consumption-first orientation grows. Without social structures that invite and expect contribution, people may drift toward lives organized around passive receipt rather than shared purpose. This drift can fuel a vicious, self-reinforcing cycle: social disconnection generates distress; distress triggers intervention; intervention is delivered as consumption rather than contribution; and shared life continues to thin. Systems become ever more active, yet increasingly unable to restore the very conditions that well-being depends upon (4). The ethical resolution is neither coercion nor indifference, but a demanding middle position in which systems preserve opportunity, communities sustain expectation, and individuals retain responsibility for whether and how they engage.

**Second, social prescribing represents an illustrative ethical bridge, not a comprehensive solution**. When practiced as an invitational mechanism — rendering opportunity visible, making access usable, and returning authorship — it exemplifies how systems can support shared life without prescribing its form (44, 46). However, when reduced to behavioral direction, lifestyle prescription, or commodified experience, it reproduces the very ethical failures it is often intended to address. The value of social prescribing lies not in the activities it connects people to, but in its refusal to decide in advance what a good life should look like. This distinction matters as social prescribing scales globally.

**Third, recovering contributory reciprocity requires structural change, not merely attitudinal shifts**. Belief in people must be embedded in commissioning decisions, evaluation frameworks, funding models, and policy design, not asserted as aspiration while systems in actuality operate on assumptions of incompetence or dependence. The recommendations in Section 6 are not technical improvements but ethical repositioning. They ask systems to relinquish control without withdrawing support, to fund infrastructure rather than only services, and to trust people with complexity even when outcomes remain uncertain.

### 7.1 Limitations and future research

This paper advances contributory reciprocity as a normative framework for organizing health and social systems. As a conceptual argument, it has important limitations.

Operationalising belief remains challenging. This paper argues that systems must believe people are capable of reasoning, growth, and contribution, and that this belief must be structurally embedded rather than rhetorically asserted. Future work should develop observable indicators of system-level belief, examining design features (opt-in vs. opt-out defaults), funding priorities (relational work vs. throughput), evaluation metrics (opportunity vs. compliance), and policy language (contribution vs. participation) as markers of operational belief.

Implementation barriers require attention. This paper does not examine why systems resist contributory reciprocity, what political and economic interests sustain current failures, or how transitions might be managed. Understanding resistance, including professional incentives, market structures, and risk-averse commissioning, is essential for effective change.

Cross-cultural applicability requires investigation. This paper draws primarily on examples from high-income welfare states, including Australia, the United Kingdom, and international policy contexts. Its relevance to other contexts, including middle- and low-income countries with different welfare architectures, remains to be examined.

Despite these limitations, contributory reciprocity offers conceptual clarity for distinguishing system responsibilities from individual agency, practical guidance for policy reform, and a framework for evaluating whether interventions support shared life or manage dependence. Whether this normative reorientation translates into improved population outcomes, stronger social connection, and sustained shared life will depend on implementation, and on whether systems have the courage to believe in people's capacity to matter to others.

Two risks warrant explicit acknowledgment. First, framing contribution as ethically significant, even while rejecting productivity-based measures of worth, carries a risk of inadvertent stigmatization. People who are unable to contribute in any recognizable form at a given time, whether due to acute illness, severe disability, cognitive change, grief, or trauma, may experience this emphasis as implicitly devaluing their current circumstances. This paper argues that belief in people's capacity is not conditional on visible contribution, and that worth is inherent rather than earned. However, the gap between this normative position and how such language is received and operationalised in practice cannot be assumed to resolve automatically.

Second, the language of civic expectation and invitation is vulnerable to misappropriation. Policymakers or commissioners who retain conditionality-based assumptions may adopt the vocabulary of contributory reciprocity, expectation, invitation, and shared life while applying it coercively, effectively reproducing welfare conditionality under an ethical register. The distinction drawn here between invitation and obligation, and between expectation and mandate, is intended to guard against this. Whether it succeeds in practice will depend on the integrity of implementation and the vigilance of those who apply it.

## 8 Conclusion

The ethical resolution proposed here is neither coercion nor indifference, but a demanding middle position that refuses to treat people as objects. Systems must preserve opportunity, protect civic space, and extend genuine invitation. Communities must sustain expectation through shared norms. Individuals must retain responsibility for whether and how they engage. None of this can be automated, commodified, or administered into existence.

The question facing health and social policy is not whether systems are doing enough, but whether they have organized themselves around the right question. A system asking, “how do we support people to consume services more efficiently?” will expand indefinitely while shared life erodes. A system asking, “how do we preserve conditions under which people can matter to others?” might actually sustain what it claims to value.

Contributory reciprocity does not require new technologies or ever-expanding budgets. It requires belief: the operational, structural, embedded belief that all people can create value for others, and that systems exist to preserve the conditions under which this remains possible. Recovering this orientation will not solve every problem health and social systems face. But without it, no amount of provision, evidence, or innovation will prevent the drift toward lives organized around taking rather than giving — and the fraying of the shared life on which human flourishing depends.
